# How patients think about social responsibility of public hospitals in China?

**DOI:** 10.1186/s12913-016-1621-1

**Published:** 2016-08-11

**Authors:** Wenbin Liu, Lizheng Shi, Raymond W. Pong, Yingyao Chen

**Affiliations:** 1 School of Public Health, Key Lab of Health Technology Assessment (Ministry of Health), Collaborative Innovation Center of Social Risks Governance in Health, Fudan University, 197 Mailbox, 138 Yixueyuan Road, Xuhui District, Shanghai, 200032 China; 2 School of Public Health and Tropical Medicine, Tulane University, New Orleans, LA 70112 USA; 3 Centre for Rural and Northern Health Research and Northern Ontario School of Medicine, Laurentian University, Sudbury, ON P3E 2C6 Canada

**Keywords:** Social responsibility, Public hospital, Multilevel regression analysis, China

## Abstract

**Background:**

Hospital social responsibility is receiving increasing attention, especially in China where major changes to the healthcare system have taken place. This study examines how patients viewed hospital social responsibility in China and explore the factors that influenced patients’ perception of hospital social responsibility.

**Methods:**

A cross-sectional survey was conducted, using a structured questionnaire, on a sample of 5385 patients from 48 public hospitals in three regions of China: Shanghai, Hainan, and Shaanxi. A multilevel regression model was employed to examine factors influencing patients’ assessments of hospital social responsibility. Intra-class correlation coefficients (ICCs) were calculated to estimate the proportion of variance in the dependent variables determined at the hospital level.

**Results:**

The scores for service quality, appropriateness, accessibility and professional ethics were positively associated with patients’ assessments of hospital social responsibility. Older outpatients tended to give lower assessments, while inpatients in larger hospitals scored higher. After adjusted for the independent variables, the ICC rose from 0.182 to 0.313 for inpatients and from 0.162 to 0.263 for outpatients. The variance at the patient level was reduced by 51.5 and 48.6 %, respectively, for inpatients and outpatients. And the variance at the hospital level was reduced by 16.7 % for both groups.

**Conclusions:**

Some hospital and patient characteristics and their perceptions of service quality, appropriateness, accessibility and professional ethics were associated with their assessments of public hospital social responsibility. The differences were mainly determined at the patient level. More attention to law-abiding behaviors, cost-effective health services, and charitable works could improve perceptions of hospitals’ adherence to social responsibility.

**Electronic supplementary material:**

The online version of this article (doi:10.1186/s12913-016-1621-1) contains supplementary material, which is available to authorized users.

## Background

Corporate social responsibility (CSR) has long been a focus of attention. Bowen defines CSR as the obligations of organizations to make their policies and decisions compatible with the values of society [1], while Carroll’s definition combines economic, legal, ethical, and philanthropic characteristics [2]. In general, social responsibility expectations typically require organizations to live up to certain public expectations and protect public welfare [3].

With public services in many countries shifting from the “social goods” model to the “economic goods” model, the privatization of public services may unintentionally weaken social responsibility [3, 4] with the healthcare system being a typical example. The role of many hospitals has shifted from the delivery of essential health services to the management of scarce resources under conditions of financial constraints. As a result, hospital social responsibility is receiving attention, especially in developing countries and transitional societies [5].

As a large transitional and developing nation, China is an important case to study. Public hospitals are a critical component of the healthcare system in China. They account for two-thirds of all hospitals in the country and provided 92.4 % of outpatient services and 92.7 % of inpatient services [6, 7]. After the health reform in the 1980s, public hospitals, which used to be heavily subsidized by government, now receive only about 10 % of their operating revenue from government sources. To compensate for their loss of revenue, public hospitals are allowed to charge fees for diagnostic tests and prescribed drugs [8, 9]. Such financial imperatives may motivate doctors to provide unnecessary health services, especially high-cost services, and prescribe expensive drugs in order to generate greater revenues for their hospitals [10, 11], which made many citizens wonder whether public hospitals have lost their sense of social responsibility [10, 12].

Hospital social responsibility has also been studied in other countries. For instance, Brandao et al. pointed out that hospitals should follow general ethical standards, promote anti-discrimination policies, and engage in national or international solidarity programs to fulfill social responsibility [3]. Fottler et al. have stressed the importance of the social process that runs in parallel to the purely medical process for the sick [13]. Dharamsi et al. have emphasized that social responsibility is a moral commitment that advances the notion of a “profession” and the attendant social contract with society [14]. According to studies by Duggirala et al. and Rohini et al., social responsibility requires every hospital to fulfill the role as a facilitator of social welfare, which refers to following regulations and legal rules to meet society’s expectations, participating in activities that promote human welfare, and so on [15–17]. In short, legal, ethical, and philanthropic characteristics are the hallmarks of hospital social responsibility.

These studies provide helpful insights regarding the nature of hospital social responsibility. However, there are few empirical studies on hospital social responsibility, especially from the patients’ perspective. To improve health system performance and patient-centered health care, it is important to understand how patients view health care, including hospital care [18]. In terms of methodology, considering the fact that patients served by the same hospital tend to present similar assessments, it is more appropriate to apply multilevel analysis which makes the study of patient and hospital characteristics together feasible. By employing multilevel techniques, this study aims to understand how patients in several regions in China view hospital social responsibility, to investigate factors that influence patients’ views of social responsibility, and estimate the proportion of variance in patients’ assessment scores determined at the hospital level and the patient level.

## Methods

### Sampling design

As China is the largest developing country in the world with different regions at diverse levels of socioeconomic development, the field survey involved a multistage sampling strategy. Firstly, Shanghai, Hainan Province, and Shaanxi Province were selected from the eastern, central and western regions of China, respectively. In the second stage, 15–17 hospitals were sampled from each of the selected regions. Lastly, approximately 120 patients, fairly evenly divided between inpatients and outpatients, from each hospital were invited to participate in the survey.

### Survey instrument

Based on suggestions from experts and findings from previous studies [3, 13–17, 19–21], we defined hospital social responsibility as hospitals’ obligations to fulfill their public mandate by achieving certain legal, ethical, and philanthropic expectations. A structured questionnaire was designed to measure patients’ perceptions of hospital performance in four dimensions: (i) service quality (with four items: effective treatment, short waiting time, tidy environment and convenient procedures); (ii) appropriateness (with three items: reasonable treatment costs, appropriate physical examination and rational prescription); (iii) accessibility in the sense of making health services accessible to as many people as possible (with three items: free treatments for the poor, equal treatment of patients and providing treatment regardless of ability to pay); and (iv) professional ethics (with two items: refusal to take bribes and protection of patient privacy). Patients’ assessments of hospital social responsibility were measured by the extent to which they believed the hospital had met societal expectations regarding their public obligations. Each of the aforementioned item was measured using a 5-point Likert scale, from “1 = very dissatisfied” to “5 = very satisfied” (Additional file 1). The reliability of the questionnaire was examined by calculating Cronbach's alpha. The corrected item-total correlation, “leave-one-out” Cronbach’s alpha and the overall Cronbach's alpha for the 12 items were shown in Table 1.

**Table 1:** Reliability of the questionnaire

Items	Inpatient (*n* = 2509)		Outpatient (*n* = 2723)	
	Corrected Item-Total Correlation	Cronbach's Alpha if Item Deleted	Corrected Item-Total Correlation	Cronbach's Alpha if Item Deleted
Effective treatment	0.688	0.905	0.610	0.874
Short waiting time	0.719	0.903	0.577	0.875
Tidy environment	0.675	0.906	0.568	0.876
Convenient procedure	0.755	0.902	0.659	0.871
Reasonable treatment costs	0.717	0.903	0.635	0.872
Appropriate physical examination	0.732	0.903	0.694	0.869
Rational prescription	0.740	0.903	0.689	0.869
Treatments for free	0.681	0.905	0.644	0.871
Equal treatment of patients	0.565	0.910	0.531	0.878
Providing treatment regardless of ability to pay	0.542	0.916	0.438	0.888
Refusal to take bribes	0.698	0.905	0.415	0.883
Protection of patient privacy	0.437	0.914	0.660	0.871
Total Cronbach's Alpha	-	0.918	-	0.889

### Data collection

Field survey started in April 2011 and lasted nine months. Trained survey facilitators distributed the questionnaires to patients in the sampled hospitals after the patients had agreed to take part and signed informed consent forms. The survey facilitators explained the questions to ensure the respondents understand what they needed to do and how to do it. The survey was completely anonymous, filled out by the patients at their convenience and returned directly to the survey facilitators.

Other relevant patient information was also collected: age, sex, visit type (inpatient or outpatient) and insurance status (with five categories: the New Cooperative Medical Scheme [NCMS] for rural residents, basic medical insurance system for urban residents [URBMI], basic medical insurance system for urban employees [UEBMI], commercial insurance [CI], and the uninsured). Two hospital characteristics were also collected: hospital size (large: > 500 beds, medium: with 100–500 beds, small: <100 beds) and region (eastern, central and western).

### Statistical analysis

Descriptive analysis was used to describe patient and hospital characteristics. Dimension scores were calculated as the mean item scores per dimension of hospital performance.

Multilevel linear regression (MLwiN version 2.02) was performed to explore the association between hospital characteristics, patient characteristics, and dimension scores of health services (independent variables) and perceived hospital social responsibility (dependent variable). The rationale for using the multilevel approach in relation to the clustering effect is the belief that patients (level 1) in the same hospital (level 2) tend to be more alike in how they view hospital social responsibility. Thus, in studies that include a large number of hospitals, such as this one, multilevel approach is more robust in determining whether factors at the hospital level or at the patient level are statistically significant. Other advantages of the multilevel modeling include the ability to simultaneously examine the relative contributions of hospital-level and patient-level factors on the outcome [22, 23]. To avoid multicollinearity, the analysis used the four dimensions, instead of the 12 items, in the model. Scores of related items were combined to form a dimension score. Because the assessments of social responsibility were negatively skewed, a square root transformation was performed to ensure the appropriateness of the final linear model. Because inpatient services are distinct from outpatient services, the multilevel regression model was run for inpatients and outpatients separately. Two-sided P-levels of <0.05 were considered to be statistically significant.

Additionally, the proportion of variance in the dependent variables determined at the hospital level was estimated by calculating intra-class correlation coefficients (ICCs). The ICCs were calculated in an empty model with a random intercept at the hospital level (raw ICC) and adjusted for independent variables (adjusted ICC).

## Results

### Study sample

Table 2 presents the characteristics of the patients and the hospitals. This study involved 5385 patients from 48 hospitals. Approximately half of the respondents were male, 21.8 % were 65 years of age or older, 52.0 % were outpatients and 42.8 % were covered by UEBMI. Concerning hospital characteristics, the hospital sample consisted of 17 small, 19 medium and 12 large hospitals; 15 hospitals were in the eastern, 16 in central and 17 in western part of the country.

**Table 2:** Characteristics of sampled patients and hospitals

Characteristic	Number	Percentage (%)
Patient characteristics (5385 patients in total) ^a^		
Age		
Younger than 65 years	3933	78.2
65 years or older	1099	21.8
Sex		
Male	2505	47.1
Female	2813	52.9
Insurance		
UEBMI	2179	42.8
Other than UEBMI	2401	47.1
No insurance	517	10.1
Visit category		
Outpatient	2723	52.0
Inpatient	2509	48.0
Hospital characteristics (48 hospitals in total)		
Region		
Eastern	15	31.3
Central	16	33.3
Western	17	35.4
Hospital size ^b^		
Large	12	25.0
Medium	19	39.6
Small	17	35.4

^a^ Because of missing data, the sum of each category may not equal to the total number of patients (*n* = 5385)

^b^ Hospital size was determined by the number of beds: large = at least 500 beds; intermediate = 100–500 beds; small = under 100 beds

### Patients’ ratings of each item in health services

Table 3 presents patients' ratings of each item of inpatient and outpatient services. In both groups, all the items except “providing treatment regardless of ability to pay” had scores of more than 4.00, with 5.00 being the maximum score. The scores for the four dimensions, which were calculated as the mean item scores per dimension, were also above 4.00.

**Table 3:** Patients’ ratings of hospital performance by visit category

Dimension	Inpatient (*n* = 2509)		Outpatient (*n* = 2723)	
Items	Mean	S.D.	Mean	S.D.
**Quality**	**4.45**	**0.65**	**4.30**	**0.62**
Effective treatment	4.37	0.85	4.49	0.66
Short waiting time	4.36	0.86	4.19	0.83
Tidy environment	4.61	0.63	4.26	0.82
Convenient procedure	4.46	0.74	4.27	0.74
**Appropriateness**	**4.34**	**0.69**	**4.18**	**0.67**
Reasonable treatment costs	4.15	0.91	4.05	0.87
Appropriate examination	4.42	0.77	4.24	0.77
Rational prescription	4.45	0.74	4.25	0.79
**Accessibility**	**4.23**	**0.71**	**4.00**	**0.69**
Treatments for free	4.27	0.83	4.07	0.84
Equal treatment of patients	4.46	0.77	4.34	0.77
Providing treatment regardless of ability to pay	3.93	1.13	3.60	1.10
**Professional ethics**	**4.65**	**0.51**	**4.51**	**0.57**
Refusal to take bribes	4.52	0.68	4.67	0.64
Protection of patient privacy	4.78	0.55	4.35	0.74

^a^ The means of the dimension score were shown in bold size

### Assessments of hospital social responsibility

**Fig. 1:**
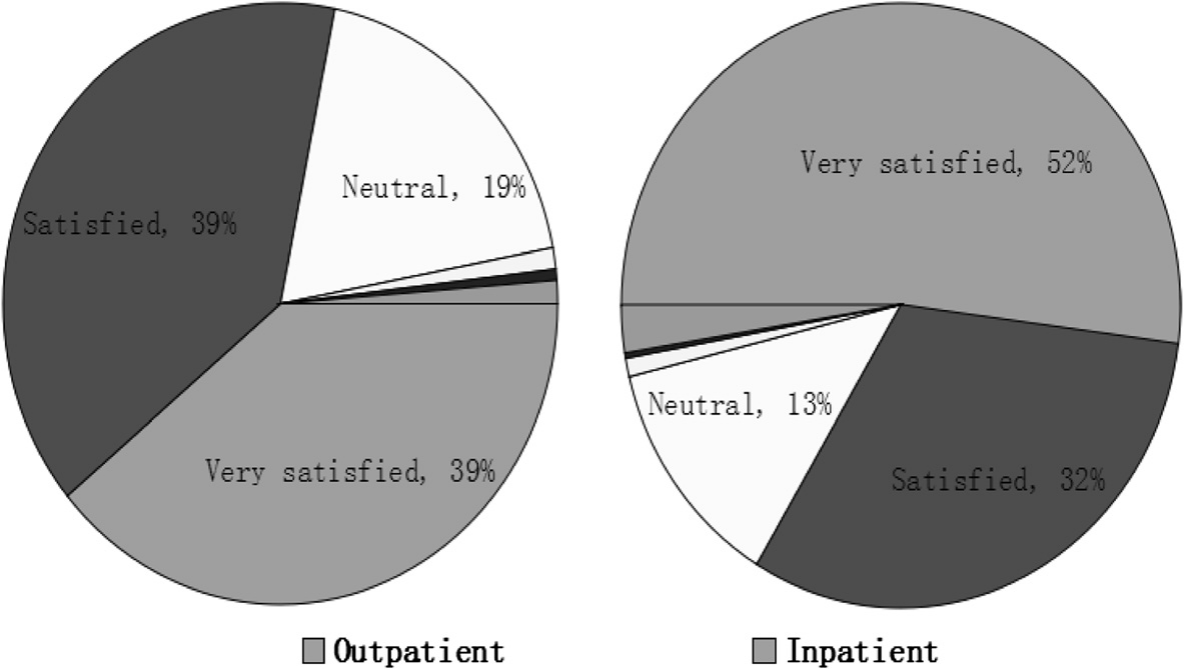
Patients’ assessments of hospital social responsibility. The figure showed the patients’ assessments of hospital social responsibility. Outpatients: Very satisfied 39.0 %, Satisfied 39.2 %, Neutral 18.9 %, Unsatisfied 1.1 %, Very unsatisfied 0.6 %, Missing 1.2 %. Inpatients: Very satisfied 52.0 %, Satisfied 31.5 %, Neutral 12.6 %, Unsatisfied 1.0 %, Very unsatisfied 0.3 %, Missing 2.6 %

A majority of the patients were satisfied or very satisfied with the social responsibility of the public hospitals studied. Those who were "very satisfied" accounted for just under 40 % of the out-patient respondents and slightly over 50 % of the inpatient respondents (see Fig. 1).

### Multilevel linear regression analysis

Table 4 presents the two final multilevel linear regression models of patient-rated social responsibility for the inpatient and outpatient groups.

**Table 4:** Final multilevel linear regression models of overall assessment on hospital social responsibility

	Inpatient (*n* = 2509)		Outpatient (*n* = 2723)	
	Coefficient	SE	Coefficient	SE
Fixed effect				
Sex (Ref: male)	0.002	0.006	−0.000	0.006
Age (Ref: < 65 years old)	−0.003	0.007	−0.019*	0.009
Insurance (Ref: UEBMI)				
Other than UEBMI	0.006	0.007	−0.007	0.009
Non-insurance	−0.006	0.011	−0.012	0.012
Perception of service quality	0.050**	0.008	0.038**	0.007
Perception of appropriateness	0.070**	0.007	0.088**	0.008
Perception of accessibility	0.096**	0.006	0.095**	0.007
Perception of professional ethics	0.044**	0.008	0.023**	0.008
Size (Ref: Small)				
Medium	0.058	0.035	0.020	0.031
Large	0.051	0.036	−0.009	0.030
Region (Ref: Eastern)				
Central	−0.065*	0.033	−0.053	0.030
Western	−0.046	0.032	−0.048	0.033
Random effect	ICC^a^	Adj ICC^b^	ICC^a^	Adj ICC^b^
Hospital, var(u_0j_)	0.182**	0.313**	0.162**	0.263**

^a^ ICC hospital level is variance hospital/total variance. Represents the amount of variance attributed to the hospital characteristics

^b^ Adjusted for the independent variables: sex, age, insurance, perception of service quality, appropriateness, accessibility and professional ethics, hospital size, region

* *P* < 0.05, ** *P* < 0.01

In both groups, the scores for the four dimensions of “service quality”, “appropriateness”, “accessibility” and “professional ethics” were all positively associated with the assessment of hospital social responsibility. With respect to patient and hospital characteristics, older outpatients gave a lower social responsibility assessment (*P* < 0.05), while inpatients in larger hospitals ranked higher (*P* < 0.05). However, no significant associations were found between social responsibility and other patient or hospital characteristics for both inpatient and outpatient groups.

The raw and adjusted ICCs (random effect) at the hospital level for inpatients and outpatients were shown in the last two rows in Table 4. The ICC represents the amount of variance determined at the hospital level. The raw ICC was 0.182 (ie, 0.006/0.033) and 0.162 (ie, 0.006/0.037), respectively, for inpatients and outpatients. After adjusted by the independent variables, the ICC rose to 0.313 (ie, 0.005/0.016) and 0.263 (ie, 0.005/0.019), respectively.

The explained variance of the independent variables was calculated by dividing the difference in variance between the empty model and the final model by the variance in the empty model. Compared to the empty model for both inpatients and outpatients, the variance associated with the hospital level was reduced by 16.7 % (ie, 0.006-0.005/0.006), almost all of which was attributable to the addition of hospital size and region. In contrast to the findings at the hospital level, the variance associated with the patient level was reduced by 51.5 % (ie, 0.033-0.016/0.033) for inpatients, and by 48.6 % (ie, 0.037-0.019/0.037) for outpatients.

## Discussion

Hospital social responsibility is of utmost importance in the current state of health system development in China. In the multilevel analysis, older outpatients and inpatients in smaller hospitals gave a lower assessment of hospital social responsibility. Other patient demographic characteristics and hospital characteristics were not found to be significantly related to hospital social responsibility. These findings were contrary to the results of many studies on patient satisfaction, which typically reported that older patients or patients in smaller hospitals tended to be more satisfied [24–28]. These differences demonstrated that there are indeed differences between perception of social responsibility and patient satisfaction. However, it is likely that the care hospitals provide still influence patients’ assessments of hospital social responsibility. In this study, the scores for “service quality”, “appropriateness”, “accessibility” and “professional ethics” have been shown to be positively associated with patients’ assessments of hospital social responsibility.

Professional ethics refers to commitments to established rules and legal obligations. Besides, law-abiding behaviors were considered as the most basic requirement of corporate social responsibility [2, 3]. Therefore, it is not surprising to find significant associations between “professional ethics” and social responsibility. Additionally, this study demonstrated that the dimension score of "service quality" was positively associated with social responsibility. This is not surprising because hospitals will not be fulfilling their social responsibility if they do not provide acceptable and effective care. However, it is worth noting that high quality health services, from the patients’ perspective, refer not merely effective clinical treatment, but also convenient services, enthusiastic attitudes, tidy environment and so on. Especially for some critically ill patients, having timely services and effective care is of great importance. If hospitals develop a patient-centered culture, take care of patients in a timely manner, help them beyond merely providing medical care, and make them feel as comfortable as possible, they are more likely to be seen as socially responsible. This is also supported by previous research, which emphasizes the interface between the social and medical processes [13].

That there is a significant relationship between "appropriateness" and hospital social responsibility is an important finding, especially in the context of current developments in the Chinese healthcare system. As noted earlier, because of the way public hospitals in China are funded, hospitals had to generate more income by providing more services, which possibly may have been perceived as less appropriate [11]. This partly explains the large number of complaints about unaffordable health services by the general public in China [7, 10].

To boost their public image and show that they are socially responsible, some businesses and private-sector organizations voluntarily engage in national or international charitable projects, such as supporting schools in Third World countries and contributing to natural disaster relief. Hospitals could do the same by helping vulnerable populations with serious or urgent health needs [3, 9]. Thus, this study has shown a positive association between “accessibility” and hospital social responsibility. Apparently, making health services accessible to those who cannot afford them was considered by those surveyed as an expression of altruism. However, this is easier said than done in China, since many public hospitals are financially stressed due to shrinking revenues from government sources. Under this context, many public hospitals had to charge more for some health services, which could have resulted in the perception of less access to some health services.. Therefore, it is not surprising that, “accessibility” had the lowest score of among the four dimensions of hospital performance.

By partitioning the variance components, the multilevel approach illustrates the relative contributions of hospital-level and patient-level factors to the variation in the assessment of hospital social responsibility. In this analysis, the ICC showed that 18.2 and 16.2 % of the total variance occurred at the hospital level for inpatients and outpatients, respectively. And for both groups, one-sixth of these were explained by hospital-level variables. These findings suggest that the differences in overall assessment of hospital social responsibility were mainly determined at the patient level. In addition, these findings also show that there still remains some unexplained variability in hospital social responsibility, which should be the focus of future studies.

### Limitations

This study has some limitations. Firstly, some patients might be reluctant to express negative views due to fear of upsetting physicians and other hospital staff, even though anonymity had been assured. This may partly explain the high ratings for hospital performance and social responsibility. Future research needs to find ways to reduce possible social desirability effects. Secondly, clustering effects were considered to exist mainly at the hospital level in this study. However, it cannot be ruled out that some variance was caused by factors at a lower level, such as at the hospital department level. If this is true, some variations at lower levels might not have been captured in this study. Thirdly, only hospitals in three regions in a very large and complex country were included in the study. This may limit the generalizability of the findings to the whole of China. Finally, the limited number of patient and hospital characteristics analyzed in this study is another limitation. There may be other characteristics - particularly hospital characteristics - that need to be investigated in future research.

## Conclusion

This study examines social responsibility of public hospitals in China from the patients’ perspective. A major finding is that how patients viewed public hospitals’ service quality, service appropriateness, service accessibility and professional ethics had a substantial impact on their assessment of a hospital’s performance with respect to social responsibility. Differences in perception of hospital social responsibility were found to be determined mainly at the patient level and, to a lesser extent, at the hospital level. This research provides some guidance to hospital administrators and physicians who wish to strengthen their hospitals’ social responsibility ratings. For instance, adherence to professional ethics (like not accepting bribes), providing high-quality services, providing appropriate services at affordable prices, making services accessible to all, and engaging in charitable acts such as providing free care for the indigent would help improve a hospital’s image of protecting public well-being and meeting societal expectations.

Although the present research concerns hospitals, in particular public hospitals in China, its finding may have implications for the study of corporate social responsibility in general. The contribution from this research to literature on corporate social responsibility is that hospitals need to be patient-centered in its planning, programming, service delivery, staff training, etc., if they want to be seen as socially responsible. Similarly, the essence of corporate social responsibility is client- or consumer-centeredness. By focusing on their clients and consumers, businesses, organizations and institutions are more likely to be successful and more likely to achieve a higher profile in relation to social responsibility.

Lastly, the situation in China may not be unique. Other transitional societies and developing nations, especially those undergoing major healthcare reform, may face challenges similar to those experienced by China. Thus, the findings of this study are also relevant to many other countries.

## Abbreviations

CSR, corporate social responsibility; ICCs, intra-class correlation coefficients; NCMS, the New Cooperative Medical Scheme for rural residents; URBMI, Basic Medical Insurance System for Urban Residents; UEBMI, Basic Medical Insurance System for Urban Employees; CI, commercial insurance.

## Additional file

Additional file 1: Questionnaire for the patients of public hospitals. The questionnaire was designed and implemented in the survey to understand patients’ opinion of the medical services, especially the view of social responsibility of this public hospital. (DOC 32 kb)
