# Supplementary material for: How patients think about social responsibility of public hospitals in China?
Source: BMC Health Serv Res. 2016 Aug 11;16:371. doi: 10.1186/s12913-016-1621-1 (PMC4982228; doi:10.1186/s12913-016-1621-1)
Supplement: Additional file 1: — Questionnaire for the patients of public hospitals. The questionnaire was designed and implemented in the survey to understand patients’ opinion of the medical services, especially the view of social responsibility of this public hospital. (DOC 32 kb) [file 12913_2016_1621_MOESM1_ESM.doc]

**Name of Hospital: __________________ ID: ___________**

**Name of investigator: _______________**

**Questionnaire for the patients of public hospitals**

To promote the improvement of hospital social responsibility, we design the questionnaire and implement the survey to understand your opinion of the medical services, especially the view of social responsibility of this public hospital. This study was approved by the ethical review committee of School of Public Health, Fudan University. It will take you a few minutes to complete the questionnaire. Taking part in this study is completely voluntary. The information you provided will be only used in the scientific research, and we promise that your privacy will be tightly protected. You do not have to participate if you don’t want to. You may also leave the study at any time. If you leave the study before it is finished, there will be no penalty to you, and you will not lose any benefits to which you are otherwise entitled. If you decide to take part in the study, you will be asked to sign in this form:

**I understand the information printed on this form. My questions so far have been answered. I agree to take part in this study.**

__________________ (Signature)

______________________ (Date)

**Socio-demographic Characteristics**

1. Gender: _________ A. Male B. Female

2. Age: _________

3. Visiting Category: _________ A. Outpatient B. Inpatient

4. Residence: _________ A. Local B. Foreign

5. Insurance: _________

A. Basic Medical Insurance System for Urban Employees （UEBMI）

B. Basic Medical Insurance System for Urban Residents （URBMI）

C. New Cooperative Medical Scheme （NCMS）

D. Commercial Insurance （CI）

E. None

6. Income: _________ A. <800 B. 800~1500 C. 1501~2500 D. > 2501

**Satisfaction**

1. Are you satisfied with the treatment effect ? _________

A. Very satisfied B. Satisfied C. Neutral D. Unsatisfied E. Very unsatisfied

1. Are you satisfied with the waiting time? _________

A. Very satisfied B. Satisfied C. Neutral D. Unsatisfied E. Very unsatisfied

1. Are you satisfied with the entire environment ? _________

A. Very satisfied B. Satisfied C. Neutral D. Unsatisfied E. Very unsatisfied

1. Are you satisfied with the procedure ? _________

A. Very satisfied B. Satisfied C. Neutral D. Unsatisfied E. Very unsatisfied

1. Are you satisfied with the treatment cost ? _________

A. Very satisfied B. Satisfied C. Neutral D. Unsatisfied E. Very unsatisfied

1. Are you satisfied with the prescription ? _________

A. Very satisfied B. Satisfied C. Neutral D. Unsatisfied E. Very unsatisfied

1. Are you satisfied with the clinical examination ? _________

A. Very satisfied B. Satisfied C. Neutral D. Unsatisfied E. Very unsatisfied

1. Are you satisfied with the measures taken to protect privacy ? _________

A. Very satisfied B. Satisfied C. Neutral D. Unsatisfied E. Very unsatisfied

1. Are you satisfied with the physicians’ behavior in bribe refusal ? _________

A. Very satisfied B. Satisfied C. Neutral D. Unsatisfied E. Very unsatisfied

1. Are you satisfied with providing treatments for free in this hospital ? _______

A. Very satisfied B. Satisfied C. Neutral D. Unsatisfied E. Very unsatisfied

1. Do you think the patient are treated equally in this hospital ? _________

A. Always B. Usually C. Sometimes D. Seldom E. Never

1. Do you think the hospital provide treatment regardless of patients’ ability to pay ? _________

A. Always B. Usually C. Sometimes D. Seldom E. Never

1. Generally speaking, How do you think about the social responsibility of this hospital ? _________

A. Very good B. Good C. Medium D. Bad E. Very bad

**That is all. Thank you for your participation!**
